# Severe anaphylaxis following off-label rectal use of injectable diazepam in a febrile seizure: a case report and clinical implications

**DOI:** 10.1186/s12245-026-01123-2

**Published:** 2026-01-20

**Authors:** Ninh Xuan Nguyen, Ngoc Tien Pham, Huong Thi Thanh Le, Quoc Viet Tran, Hang Ngoc Thuy Tran, Thi Kim Thanh Vo, Chuong Thi Ngoc Dang

**Affiliations:** 1 Emergency Department, Vinmec Central Park International Hospital, Vinmec Healthcare System, Ho Chi Minh City, Vietnam; 2 Pediatrics Department, Vinmec Central Park International Hospital, Vinmec Healthcare System, Ho Chi Minh City, Vietnam

**Keywords:** Febrile seizure, Rectal diazepam, Propylene glycol, Pediatric anaphylaxis, Off-label use, Excipient hypersensitivity, Emergency medicine, Case report

## Abstract

**Background:**

Off-label rectal administration of intravenous diazepam is commonly practiced for acute seizure management in children when FDA-approved rectal gels (e.g., Diastat^®^ AcuDial) are inaccessible—particularly in low-resource settings like Vietnam. However, such injectable formulations often contain excipients not validated for mucosal use, including propylene glycol and sodium benzoate, which may induce hypersensitivity reactions. Reports of anaphylaxis in this context remain exceedingly rare.

**Case presentation:**

We describe a case of suspected grade III anaphylaxis in a 14-month-old girl with complex febrile seizure who received 2 mg rectal diazepam (injectable formulation, Vidipha, Vietnam). Within five minutes of administration, she developed generalized urticaria, apnea, cyanosis, and hypotonia. Emergency treatment included intramuscular epinephrine, corticosteroids, antihistamines, and mechanical ventilation. She made a full recovery. The diazepam preparation used contained approximately 40% propylene glycol and also included sodium benzoate—both of which have been reported as potential triggers of non–IgE-mediated hypersensitivity reactions, particularly when delivered via mucosal routes.

**Conclusion:**

This case underscores the potential for life-threatening adverse events following off-label rectal use of injectable diazepam. Emergency clinicians should be aware of excipient-related risks and prioritize safer alternatives such as intranasal midazolam. There is an urgent need for regulatory efforts to improve access to mucosal-safe, pediatric-appropriate benzodiazepine preparations in resource-limited healthcare systems.

## Introduction

Febrile seizures are the most common neurologic disorder in children, affecting 2–5% of those aged 6 months to 5 years [1]. While typically benign, complex febrile seizures—characterized by focal onset, prolonged duration, or recurrence within 24 h—require prompt pharmacologic treatment to prevent complications such as status epilepticus or aspiration [2, 3].

Benzodiazepines are the cornerstone of acute seizure management. Rectal diazepam, especially via injectable formulations repurposed for mucosal use, is often employed off-label in prehospital or emergency settings due to its rapid absorption and utility when IV access is unavailable [4]. The only FDA-approved rectal diazepam (Diastat^®^ AcuDial) is a gel formulation with excipients optimized for mucosal safety and absorption [4]. However, in many low- and middle-income countries—including Vietnam—Diastat^®^ remains inaccessible or unaffordable, leading to widespread off-label rectal use of injectable diazepam in pediatric emergency care [5, 6].

In Vietnam, Diastat^®^ is not marketed, and intranasal midazolam—despite being a safer alternative—is rarely stocked in public hospitals. Therefore, rectal administration of injectable diazepam remains a practical solution for many clinicians. One study at Vietnam National Children’s Hospital reported that 13.1% of pediatric emergency seizures were treated with rectal diazepam [7].

This off-label practice poses safety concerns. Injectable diazepam contains excipients like propylene glycol, ethanol, and sodium benzoate, which are not evaluated for mucosal use and have been linked to local irritation, systemic toxicity, and rare hypersensitivity reactions [8–11].

Despite these risks, adverse reactions remain underreported. We describe a rare case of grade III anaphylaxis following off-label rectal use of parenteral diazepam in a toddler, underscoring the need for institutional guidance and regulatory oversight in pediatric seizure emergencies.

## Case presentation

A previously healthy 14-month-old girl (11 kg, 80 cm) presented to the emergency department (ED) with generalized tonic-clonic seizures and perioral cyanosis following one day of febrile illness. She had no history of allergy, atopy, or chronic disease; family history was unremarkable for epilepsy or hypersensitivity.

The seizure began at home, described as generalized stiffness, eye-rolling, and cyanosis. Prior to arrival, her temperature was 39.5 °C. On arrival (12:25 PM, 29 May 2025), she was actively seizing with SpO₂ at 75% on room air. Oxygen was administered via nasal cannula (6 L/min). As IV access was not immediately obtainable, a rectal dose of diazepam 2 mg (0.2 mL of injectable diazepam 10 mg/2 mL, Vidipha, Vietnam) containing 40% propylene glycol and sodium benzoate was given using a syringe and soft cannula.

Within five minutes, she developed generalized urticaria, erythema, perioral cyanosis, and apnea. Based on the Ring and Messmer classification, a diagnosis of grade III anaphylaxis was made, given the presence of acute apnea requiring airway intervention, severe hypoxemia, hypotension (60/35 mmHg), and bradycardia. A Code White was activated. Emergency treatment included intramuscular adrenaline (0.01 mg/kg), IV methylprednisolone, and diphenhydramine. Due to ongoing apnea and hypoxia, she was intubated (ETT 4.0, depth 12 cm) and ventilated (AC/PC, PIP 18 cmH₂O, PEEP 6 cmH₂O, FiO₂ 40%).

Laboratory tests showed mild lymphocytosis, CRP 1.66 mg/L, and slightly elevated AST (78.4 U/L) and ALT (54.0 U/L). Electrolytes, glucose, and lactate were normal. CSF was clear with 5 WBCs/mm³, glucose 5.26 mmol/L, protein 0.15 g/L; Gram stain, culture, and multiplex PCR were negative. Dengue NS1 antigen and blood cultures were also negative.

**Fig. 1:**
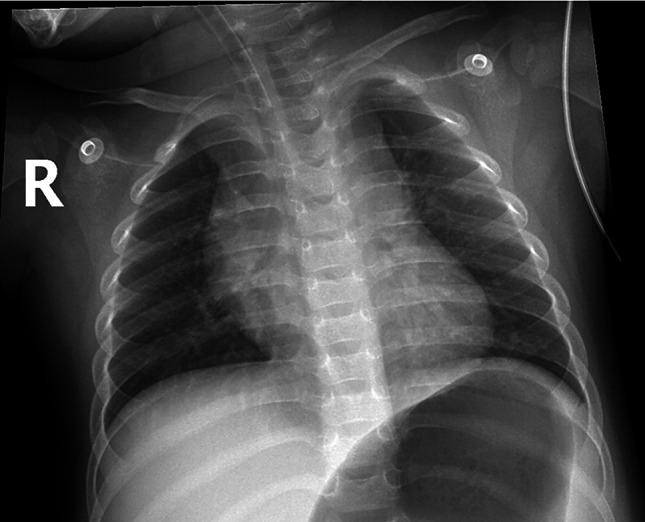
Portable chest radiograph on admission (29/05/2025). Portable anteroposterior chest radiograph demonstrating bilateral perihilar bronchial wall thickening and peribronchial cuffing, suggestive of acute bronchitis or early bronchopneumonia. No evidence of focal consolidation or pleural effusion is noted. The radiopaque silhouette of an endotracheal tube is present, with its tip approximately 2 cm above the carina—consistent with appropriate placement for mechanical ventilation

A chest X-ray (Fig. 1) revealed perihilar bronchial wall thickening, suggestive of acute bronchitis. A presumptive diagnosis of upper respiratory tract infection was made, and empirical IV amoxicillin–clavulanate was initiated.

Two hours later, the patient resumed spontaneous breathing and was extubated. She remained hemodynamically stable with no further seizures. Observation in the pediatric ward over 72 h showed no recurrence of fever, neurologic deficits, or hypersensitivity. She was discharged on 02 June 2025 with the final diagnoses: complex febrile seizure, upper respiratory tract infection, and suspected grade III anaphylaxis to rectal diazepam (injectable formulation).

Table 1 summarizes the patient’s clinical course, interventions, and outcomes from admission through discharge.

**Table 1:** Chronological summary of clinical course, interventions, and outcomes in a 14-month-old girl with febrile seizure and suspected anaphylaxis following rectal diazepam administration

Time (dd/mm/yyyy)	Clinical status	Vital signs (HR / BP / SpO₂)	Interventions	Outcomes / Notes
12:25–29/05/2025 (Seizure onset)	Generalized tonic-clonic seizure; perioral cyanosis	HR 180 bpm; BP not measured; SpO₂ 75% (room air)	Oxygen 6 L/min via nasal cannula; rectal diazepam 2 mg (Vidipha inj.); lateral positioning	Seizure persisted; developed urticaria, apnea, worsening cyanosis within 5 min
12:30 (Anaphylaxis onset)	Rash, apnea, hypotonia, bradycardia	HR 65 bpm; BP 60/35 mmHg; SpO₂ 62%	IM epinephrine 0.11 mg (0.01 mg/kg); IV methylprednisolone; IV diphenhydramine; bag-mask ventilation	Transient stabilization; minimal spontaneous respiration
12:40 (Progression)	Persistent apnea; no spontaneous breathing	HR 90 bpm; BP 75/40 mmHg; SpO₂ 84% (with bag-mask)	Endotracheal intubation (ETT 4.0, depth 12 cm); mechanical ventilation in assist-control pressure control (AC/PC) mode: PIP 18 cmH₂O, PEEP 6 cmH₂O, FiO₂ 40%	SpO₂ improved; urticaria resolved gradually
13:30 (Recovery)	Partial spontaneous breathing	HR 110 bpm; BP 85/45 mmHg; SpO₂ 95%	Switched to synchronized intermittent mandatory ventilation (SIMV) mode (PS 8, PI 14, PEEP 6); FiO₂ 40%	Stable ventilation; symmetric breath sounds
14:00	Fully alert; breathing spontaneously	HR 105 bpm; BP 90/50 mmHg; SpO₂ 98%	Extubated; oxygen 3 L/min via nasal cannula	No respiratory distress
14:30	Post-stabilization evaluation	HR 100 bpm; BP 90/50 mmHg; SpO₂ 99%	Lumbar puncture, CSF analysis, blood glucose, multiplex PCR, chest X-ray	CSF normal; X-ray: bronchial wall thickening
30–31/05/2025	Afebrile; mild URTI symptoms	HR 95–100 bpm; BP stable; SpO₂ >97%	Supportive care; oral paracetamol; feeding tolerated	No further seizures or allergic signs
01–02/06/2025 (Discharge)	Fully recovered	HR 90–95 bpm; BP 95/60 mmHg; SpO₂ 99%	Completed amoxicillin–clavulanate; discharge plan implemented	Discharged home on 02/06/2025

## Discussion

This case describes a rare but critical episode of suspected anaphylaxis following the off-label rectal administration of injectable diazepam in a febrile infant with first-time complex febrile seizure. It raises important issues regarding off-label drug use, the immunogenic risk of pharmaceutical excipients, and emergency seizure management protocols in pediatric settings, particularly in low-resource environments where FDA-approved formulations like Diastat may not be readily available. According to the Ring and Messmer classification, grade III anaphylaxis is defined by life-threatening respiratory compromise and/or cardiovascular instability, both of which were present in this patient.

### Off-label rectal use of injectable diazepam: practice vs. policy

Rectal diazepam is a well-established option for managing acute seizures when IV access is delayed [3, 4]. Diastat^®^ AcuDial is the only formulation approved by the U.S. FDA for rectal use, featuring pH buffers and excipient concentrations optimized for mucosal safety [4].

In contrast, injectable diazepam (e.g., Diazepam 10 mg/2 mL by Vidipha, Vietnam) is formulated for intravenous or intramuscular use and contains excipients such as propylene glycol (~ 40%), ethanol (10–15%), sodium benzoate, and benzyl alcohol (1.5%)—none of which are optimized for rectal administration. These components have limited or unvalidated mucosal safety data, especially when used at high concentrations. Rectal use of such formulations is off-label and has not been formally evaluated for this route [5, 12].

Nonetheless, such practice is widespread in Vietnam’s pediatric emergency settings, driven by limited access to rectal gel or intranasal midazolam [7]. While national guidelines remain silent, some hospitals allow this use under pragmatic protocols.

This disconnect between approved indications and actual clinical practice raises serious safety concerns, especially in vulnerable pediatric populations during emergencies.

### Enhanced risk from rectal absorption and enzyme deficiency

Rectal administration provides rapid systemic absorption by bypassing hepatic first-pass metabolism via the inferior and middle rectal veins [13]. While this supports fast seizure control with lipophilic drugs like diazepam, it also heightens systemic exposure and risk of adverse reactions.

Crucially, rectal mucosa exhibits low expression of metabolizing enzymes such as cytochrome P450s and UDP-glucuronosyltransferases, unlike hepatic or intestinal tissue. This limits local detoxification, particularly of excipients like propylene glycol and ethanol that require hepatic processing to mitigate toxicity [8, 9].

Additionally, injectable diazepam formulations (e.g., Vidipha) are aqueous-alcoholic solutions lacking the viscosity and buffering agents of rectal gels like Diastat^®^ [12]. This enables faster transmucosal diffusion, resulting in higher peak plasma levels of both diazepam and excipients [13]. Such pharmacokinetics may trigger mast cell degranulation or IgE-independent hypersensitivity responses [11].

In our patient, anaphylaxis occurred within five minutes of administration, suggesting that rectal delivery not only accelerates drug absorption but also facilitates rapid immunologic reaction. These findings underscore the need to assess excipients—not just the active drug—when considering off-label mucosal routes.

Table 2 compares the excipient profiles of Vidipha diazepam with Diastat^®^ gel.

**Table 2:** Comparative excipient composition and formulation characteristics of rectal diazepam gel (Diastat^®^ AcuDial) and injectable diazepam (Vidipha) used off-label via the rectal route excipients listed are based on official product labeling; where exact concentrations are not specified in the package insert, approximate ranges are reported according to standard injectable diazepam formulations. Differences in formulation design and excipient profiles May influence mucosal absorption, tolerability, and the risk of adverse reactions

Component / property	Diastat^®^ AcuDial (FDA-approved for rectal use)	Diazepam injection Vidipha (intended for IV/IM; used off-label rectally)	Clinical considerations
Active ingredient	Diazepam 5 mg/mL	Diazepam 5 mg/mL	Same active drug; formulation differs.
Propylene glycol	Present *(amount not specified in FDA label)* [15]	Present (~ 40%) [16]	Solvent associated with toxicity at high exposure; potential non–IgE-mediated reactions reported with excipients [7,8,13].
Ethanol	Present *(FDA label lists alcohol; concentration specified on label)* [15]	Present (as listed in package insert; concentration may vary by manufacturer) [16]	Mucosal irritation and systemic effects possible; rapid mucosal absorption may increase exposure.
Sodium benzoate / benzoic acid system	Present (listed in FDA label) [15]	Present (listed in package insert) [16]	Benzoates have been linked to histamine release/urticaria in susceptible individuals [13].
Benzyl alcohol	Presen**t** *(listed in FDA label)* [15]	Present (~ 1.5%) [16]	Preservative; caution in infants/young children; potential hypersensitivity in susceptible individuals.
Thickening / gel matrix	Gel base with hydroxypropyl methylcellulose (hypromellose) [15]	Not applicable	Gel base improves rectal retention and controlled absorption.
Formulation type	Rectal-specific gel, FDA-approved [15]	Aqueous–alcoholic injectable solution (parenteral formulation) [16]	Off-label rectal use of injectable solution may alter absorption kinetics and tolerability.
Buffer / pH information	Buffered system described in labeling (rectal product) [15]	Not specified in package insert [16]	Buffering may affect mucosal tolerability; lack of stated pH data limits assessment.

Abbreviations: IV, intravenous; IM, intramuscular; FDA, Food and Drug Administration

Sources for composition: Diastat® label [15]; Vidipha package insert [16]. Safety/immunologic discussion supported by [7,8,13].

### Immunologic triggers from propylene glycol and sodium benzoate

Propylene glycol (PG) and sodium benzoate—common excipients in parenteral diazepam formulations—are increasingly recognized as potential triggers for hypersensitivity reactions, including both IgE- and non-IgE-mediated anaphylaxis [11].

### Propylene glycol (PG)

PG is used at concentrations up to 40% in injectable diazepam. Although considered safe for IV use in adults, evidence suggests heightened toxicity and immunogenicity in pediatric populations [8, 9]. Adverse effects include metabolic acidosis, CNS depression, renal dysfunction, and hemolysis—particularly when rapidly absorbed via mucosal surfaces.

Notably, PG may act as a non-IgE-mediated anaphylactogen. A systematic review by González‑Rodríguez et al. (2022) reported PG-induced mast cell degranulation without prior sensitization, especially via non-parenteral routes like rectal or intrathecal administration [11]. In vitro studies also show that high PG concentrations (> 35%) can directly activate the MrgprX2 receptor on mast cells, provoking pseudo-allergic reactions that mimic classic anaphylaxis [11].

High concentrations of propylene glycol, such as those reported in certain injectable diazepam formulations [12], may theoretically increase the risk of non–IgE-mediated hypersensitivity through rapid mucosal absorption. When combined with other excipients with histamine-releasing potential, this may contribute to acute systemic reactions. However, in the absence of formal allergy testing, including skin testing or controlled provocation, definitive attribution to diazepam or any specific excipient cannot be established [8, 11–13].

### Sodium benzoate

 Sodium benzoate, included in some Vietnamese diazepam formulations such as Vidipha, has been linked to hypersensitivity reactions. While traditionally viewed as inert, recent reviews highlight its potential to cause histamine release, contact urticaria, and worsening of atopic conditions in susceptible individuals [11].

 Importantly, both sodium benzoate and propylene glycol are also present in Diastat^®^ AcuDial, the FDA-approved rectal diazepam formulation [14]. However, Diastat^®^ is specially formulated with excipient concentrations optimized for mucosal safety, unlike injectable diazepam solutions intended for IV/IM use.

In this case, the patient had no known prior exposure to diazepam. However, prior exposure to propylene glycol cannot be excluded, given its widespread presence in pharmaceuticals and consumer products.

 This case emphasizes the need to consider not only the active drug but also excipient compatibility when medications are used via non-approved routes, especially in resource-limited settings [5, 11, 14].

### Regulatory perspective and need for updated guidelines

Neither the WHO Model List of Essential Medicines for Children [15] nor UpToDate [16] recommends the rectal administration of IV diazepam. Instead, intranasal midazolam or buccal lorazepam is preferred when IV access is not available [3, 13]. While these routes may not be widely accessible in low-resource settings, transitioning away from unapproved practices is crucial.

Although Vietnamese formularies may permit rectal use of injectable diazepam in emergencies, this case highlights the urgent need to re-evaluate such protocols, particularly concerning excipient safety and pharmacologic justification [7].

Despite endorsement by international guidelines, intranasal-specific midazolam products are not commercially available in Vietnam [3, 17]. Injectable forms are often repurposed off-label for intranasal use, but lack of atomizers and inconsistent delivery may limit efficacy [17].

Likewise, Diastat^®^—the only FDA-approved rectal diazepam—is unavailable on the Vietnamese market [14]. As a result, clinicians often rely on intravenous diazepam formulations for rectal use, despite concerns about excipient-related toxicity and mucosal safety.

These constraints underline the need for national strategies to improve access to mucosal-safe benzodiazepines. Solutions may include local registration of intranasal/rectal products, technology transfer for domestic production, price negotiations, or public subsidies. Such policies could bridge the gap between evidence-based care and practice realities, enhancing pediatric seizure management across Vietnam.

### Study limitations

This report describes a single-patient case, limiting its generalizability. The diagnosis of anaphylaxis relied on clinical observation alone; confirmatory tests such as serum tryptase or allergen-specific IgE were not performed, as they are often unavailable in acute pediatric settings.

Due to ethical constraints, re-challenge testing or component-specific allergen identification was not conducted. These limitations preclude definitive etiologic confirmation but reflect real-world diagnostic challenges in emergency care.

### Clinical perspective

While this event occurred in a tertiary hospital with timely intervention, similar reactions in lower-resource settings may have worse outcomes. Many emergency departments in low- and middle-income countries continue off-label rectal use of injectable diazepam due to lack of access to Diastat^®^ or intranasal midazolam [10, 17].

This case underscores the potential for life-threatening reactions from unapproved administration routes. It calls for critical reassessment of institutional formularies and risk-benefit evaluation of mucosal drug use—particularly in pediatric care—based on pharmacologic safety rather than availability alone.

### Future research directions

Further research is warranted to fill knowledge gaps regarding off-label rectal use of injectable diazepam. Comparative pharmacokinetic studies between injectable diazepam and Diastat^®^ could elucidate differences in absorption and systemic exposure [5]. National multicenter surveillance in Vietnam may help quantify adverse reactions linked to excipients, especially among children.

Additionally, in vitro studies exploring mast cell activation via the MrgprX2 pathway by propylene glycol or sodium benzoate may clarify mechanisms underlying non-IgE-mediated hypersensitivity [11]. These findings would support safer formulation design and informed regulatory action.

## Conclusion

This case illustrates the serious risks associated with rectal administration of injectable diazepam in pediatric emergencies. While off-label practices often arise from access limitations, their safety should not be assumed. Severe hypersensitivity reactions, including anaphylaxis, may be associated with formulation excipients; however, definitive causal attribution cannot be established in the absence of formal allergy testing.

Clinicians should favor evidence-based options like intranasal midazolam and advocate for mucosal-appropriate formulations in hospital formularies. Training in hypersensitivity recognition and regular pharmacovigilance reviews are equally essential.

This report reinforces the global need for harmonized treatment protocols and wider availability of approved, excipient-safe therapies for pediatric seizure emergencies, particularly in resource-limited settings.

## Learning points

Avoid rectal administration of injectable diazepam formulations not intended for mucosal use.

Recognize the potential risk of anaphylaxis related to excipients such as propylene glycol and sodium benzoate.

Prefer intranasal midazolam as a safer, guideline-recommended alternative in pediatric seizures.

Update formularies and policies to reflect excipient safety and route-specific risks.

Train clinical staff in early recognition and response to drug-induced hypersensitivity.

Promote global attention to route-related risks in low-resource seizure care.

Understand that such risks are relevant across many low-resource settings, highlighting the global imperative for safe and equitable seizure care.

## Data Availability

All data generated or analyzed during this case report are included within the published article. Additional supporting materials—such as anonymized laboratory results, imaging, and treatment timeline—can be made available upon reasonable request to the corresponding author, provided that local privacy and data-sharing regulations are followed.

## Declarations

### Authors' information (optional)

Dr. Ninh Xuan Nguyen is Head of the Emergency Department at Vinmec Central Park International Hospital (Ho Chi Minh City, Vietnam). His academic interests include pediatric emergency medicine, adverse drug reaction monitoring, and real-world safety of off-label therapies.
